# Supplementary material for: Dual Inoculation with Rhizophagus irregularis and Bacillus megaterium Improves Maize Tolerance to Combined Drought and High Temperature Stress by Enhancing Root Hydraulics, Photosynthesis and Hormonal Responses
Source: Int J Mol Sci. 2023 Mar 8;24(6):5193. doi: 10.3390/ijms24065193 (PMC10049376; doi:10.3390/ijms24065193)
Supplement: Supplementary file 1 [file ijms-24-05193-s001.zip › Table S1.pdf]

Table S1. Pearson correlation coefficient matrix of twenty physiological and hormonal plant traits measured: Relative Electrolyte Leakage (REL), Shoot Dry Weight (SDW), Shoot to Root Ratio (S:R), Root Dry Weight (RDW), Photosystem II Efficiency (PSII Eff.), Relative Water Content (RWC), Osmotic root hydraulic conductivity (Lo), hydrostatic root hydraulic conductivity (Lpr), Abscisic Acid (ABA), Indoleacetic Acid (IAA), Salicylic Acid (SA), Jasmonic Acid (JA), Jasmonate Isoleucine (JA-Ile), Aquaporin genes *ZmPIP1;3*, *ZmPIP2;2*, *ZmTIP1;1* from maize and *GintAQPF1* from *Rhizophagus irregularis*, net photosynthetic rate (A_n), stomatal conductance (g_s) and intrinsic water use efficiency (WUE_i). Correlation p value is expressed as follows: <0.05 blue, <0.01 orange, <0.001 purple and <0.0001 red color.

	REL	SDW	S:R	RDW	PSII Eff.	RWC	Lo	Lpr	ABA	IAA	SA	JA	Ja-Ile	*ZmPIP1;3*	*ZmPIP2;2*	*ZmTIP1;1*	*GintAQPF1*	A_n	g_s	WUE_i
**REL**	1																			
**SDW**	-0,943	1																		
**S:R**	0,0313	0,2045	1																	
**RDW**	-0,6674	0,5262	-0,7061	1																
**PSII Eff.**	-0,9491	0,9624	0,0943	0,6041	1															
**RWC**	-0,8221	0,7815	-0,0481	0,5185	0,7169	1														
**Lo**	-0,5862	0,7383	0,6766	-0,0747	0,678	0,5406	1													
**Lpr**	-0,5251	0,6712	0,7984	-0,1921	0,6028	0,3904	0,8895	1												
**ABA**	0,2903	-0,1083	0,7226	-0,6392	-0,1009	-0,4898	0,4603	0,4841	1											
**IAA**	0,7463	-0,6126	-0,0744	-0,3129	-0,679	-0,7206	-0,5973	-0,548	0,105	1										
**SA**	0,1036	-0,0889	-0,3163	0,2453	-0,1686	-0,2178	-0,5813	-0,4054	-0,4535	0,6366	1									
**JA**	0,5714	-0,5677	-0,35	-0,0691	-0,562	-0,6879	-0,7864	-0,7007	-0,141	0,8737	0,7827	1								
**Ja-Ile**	-0,8743	0,8767	0,0423	0,5339	0,8935	0,6518	0,5648	0,4168	-0,1644	-0,5137	-0,0113	-0,3162	1							
ZmPIP1;3	0,1686	-0,0028	0,6488	-0,5182	-0,0215	-0,1327	0,6218	0,5407	0,8385	-0,1666	-0,7477	-0,5334	-0,2151	1						
ZmPIP2;2	-0,6184	0,4711	-0,6969	0,9335	0,5505	0,4297	-0,0179	-0,2324	-0,4683	-0,2872	0,1308	-0,0488	0,5617	-0,3853	1					
ZmTIP1;1	-0,3542	0,3947	0,2351	0,0546	0,3088	0,2286	0,5948	0,3971	0,4174	-0,2716	-0,3443	-0,3865	0,337	0,4903	0,3236	1				
GintAQPF1	-0,067	0,2592	0,5909	-0,2674	0,3289	0,0378	0,6602	0,5838	0,6376	-0,2668	-0,6548	-0,5257	0,1165	0,7505	-0,2824	0,0724	1			
**A_n**	-0,9766	0,9594	-0,0019	0,674	0,9834	0,7764	0,6528	0,5441	-0,1735	-0,7093	-0,1755	-0,5873	0,8822	-0,0432	0,6457	0,4008	0,2236	1		
**g_s**	-0,9799	0,9449	-0,1119	0,755	0,9426	0,8186	0,513	0,4427	-0,3717	-0,6341	0,0207	-0,4798	0,8587	-0,2417	0,6766	0,2883	0,0401	0,97	1	
**WUE_i**	0,8713	-0,7967	0,3062	-0,8151	-0,7808	-0,7814	-0,2106	-0,2108	0,6545	0,4771	-0,2908	0,2668	-0,7202	0,5379	-0,6544	-0,0129	0,217	-0,814	-0,9283	1
