# Supplementary material for: Dual Inoculation with Rhizophagus irregularis and Bacillus megaterium Improves Maize Tolerance to Combined Drought and High Temperature Stress by Enhancing Root Hydraulics, Photosynthesis and Hormonal Responses
Source: Int J Mol Sci. 2023 Mar 8;24(6):5193. doi: 10.3390/ijms24065193 (PMC10049376; doi:10.3390/ijms24065193)
Supplement: Supplementary file 1 [file ijms-24-05193-s001.zip › Table S2.pdf]

	Two-way ANOVA									Normality distribution test					
Factor/ Parametric *Statistics*/ Parameter	Abiotic stress (AS)			Inoculation (I)			AS x I			Normal distribution Shapiro-Wilk test		Logaritmic distribution D Kolmogorov test		Johnson distribution Shapiro-Wilk test	
	df	F	p value	df	F	p value	df	F	p value	W	p value	D	p value	W	p value
REL	1	382.673	<0.0001	3	19.2547	<0.0001	3	9.5742	<0.0001	-	-	-	-	0.972733	0.0848
SDW	1	48.0494	<0.0001	3	9.3017	<0.0001	3	1.4267	0.2388	0.981984	ns	-	-		
RDW	1	36.0097	<0.0001	3	3.0226	0.0327	3	1.8982	0.1340	-	-	0.041691	0.1500		
S:R	1	2.8497	0.0946	3	7.3086	0.0002	3	1.4601	0.2303	0.986494	0.3763	-	-		
WUE_i	1	53.9231	<0.0001	3	0.0732	0.9740	3	0.3990	0.7544	-	-	0.110189	0.1491		
RWC	1	55.9468	<0.0001	3	15.3594	<0.0001	3	0.8843	0.4550	0.985688	0.6765	-	-		
L_o	1	5.7930	0.0208	3	58.1622	<0.0001	3	5.4599	0.0031	0.955483	0.0663				
Lpr	1	1.9049	0.1771	3	19.2173	<0.0001	3	0.1266	0.9437	0.957648	0.1391				
ABA	1	24.2111	<0.0001	3	8.5624	0.0002	3	3.3825	0.0285	-	-	0.115256	0.1464	-	-
IAA	1	7.3686	0.0153	3	7.0598	0.0031	3	4.0082	0.0264	-	-	-	-	0.962592	0.4927
SA	1	13.2080	0.0009	3	9.8662	<0.0001	3	5.1073	0.0050	-	-	-	-	0.978252	0.5947
JA	1	1.0077	0.3225	3	19.8810	<0.0001	3	3.6359	0.0223	-	-	0.112350	0.1500	-	-
ZmPIP1;3	1	71.4569	<0.0001	3	39.4721	<0.0001	3	7.3224	0.0012	0.973962	0.6151	-	-	-	-
ZmPIP2;2	1	42.5030	<0.0001	3	8.4194	0.0005	3	8.7050	0.0004	-	-	0.152359	0.0577	-	-
ZmTIP1;1	1	0.3135	0.5807	3	1.6450	0.2054	3	7.9024	0.0008	0.959804	0.2713	-	-	-	-
GintAQPF1	1	18.0679	0.0014	1	1.2534	0.2867	1	3.9869	0.0712	0.897267	0.0727	-	-	-	-
Non parametric statistics / parameter	Abiotic stress (AS)			Microorganism inoculation (I)			ASxI								
	df	Xi^2	p value	df	Xi^2	p value	df	Xi^2	p value						
PSII efficiency	1	41.6261	<0.0001	3	10.3813	0.0156	3	54.5613	<0.0001						
A_n	1	30.4647	<0.0001	3	7.8921	0.0483	3	38.2775	<0.0001						
g_s	1	35.2653	<0.0001	3	3.6607	0.3005	3	40.0646	<0.0001						
Ja-Ile	1	11.5592	0.0007	3	3.5712	0.3116	3	24.2018	0.0010						

Table S2. Statistical results of two factors and their interaction for twenty physiological, molecular and hormonal plant traits measured: Relative Electrolyte Leakage (REL), Shoot Dry Weight (SDW), Shoot to Root Ratio (S:R), Root Dry Weight (RDW), Photosystem II Efficiency (PSII Eff.), Relative Water Content (RWC), Osmotic root hydraulic conductivity (Lo), hydrostatic root hydraulic conductivity (Lpr), Abscisic Acid (ABA), Indoleacetic Acid (IAA), Salicylic Acid (SA), Jasmonic Acid (JA), Jasmonate Isoleucine (JA-Ile), Aquaporin genes *ZmPIP1;3*, *ZmPIP2;2*, *ZmTIP1;1* from maize and *GintAQPF1* from *Rhizophagus irregularis*, net photosynthetic rate (A_n), stomatal conductance (g_s) and intrinsic water use efficiency (WUE_i). Two factors: Abiotic stress, with two levels: non stress and combined drought+high temperature; and inoculation factor, with four levels: control (no-microorganism inoculated), *Bacillus megaterium* (Bm), *Rhizophagus irregularis* (AM), and AM+Bm combination. Degree of fredom (df), F reason and p value of two-way Anova analysis and Normality tests are shown. When necessary, variables were transformed, being the null hypothesis >0.05 -that the population is normally distributed-. After transformation, parametric analysis was performed. When after transformation, H0 persist significant, we applied Kurskal-Wallis as a non-parametric analysis, and Xi^2 and respective p value is showed in the table. For Post Hoc test on non-parametric variables, Tukey-Kramer test was performed.
