# Supplementary figures and images for: Dual Inoculation with Rhizophagus irregularis and Bacillus megaterium Improves Maize Tolerance to Combined Drought and High Temperature Stress by Enhancing Root Hydraulics, Photosynthesis and Hormonal Responses
Source: Int J Mol Sci. 2023 Mar 8;24(6):5193. doi: 10.3390/ijms24065193 (PMC10049376; doi:10.3390/ijms24065193)

**S1**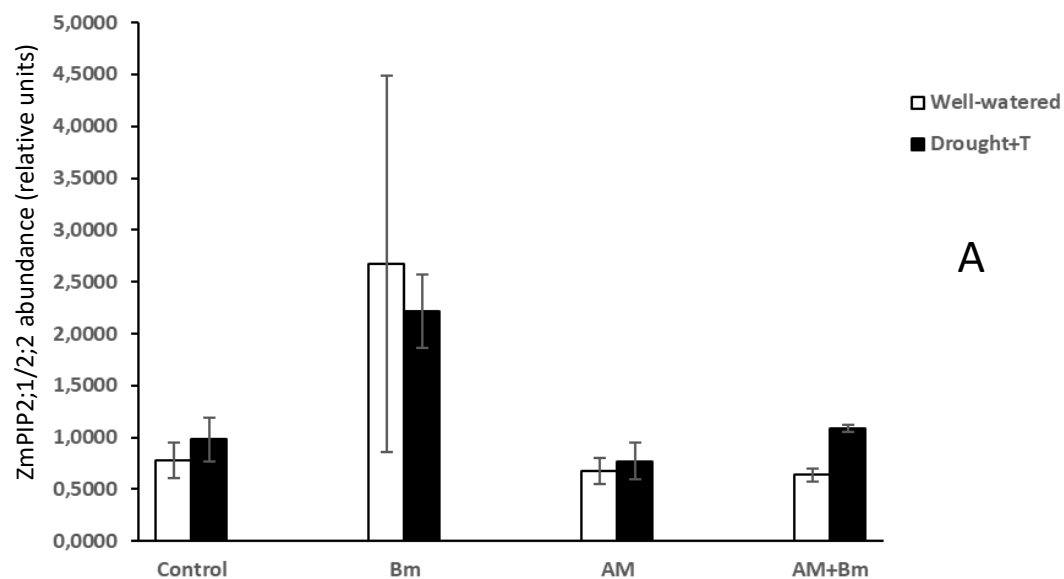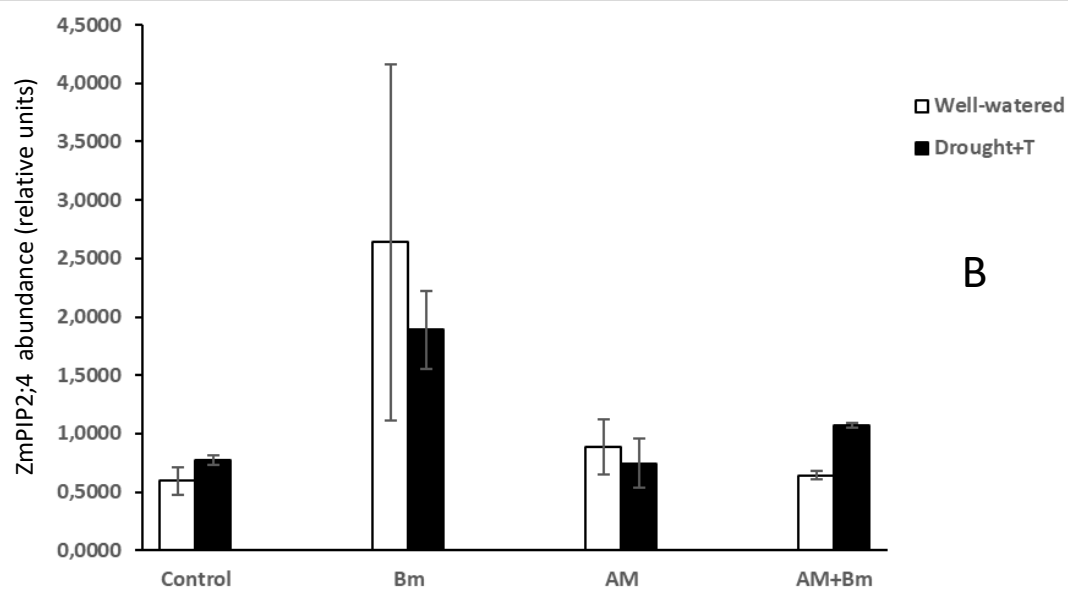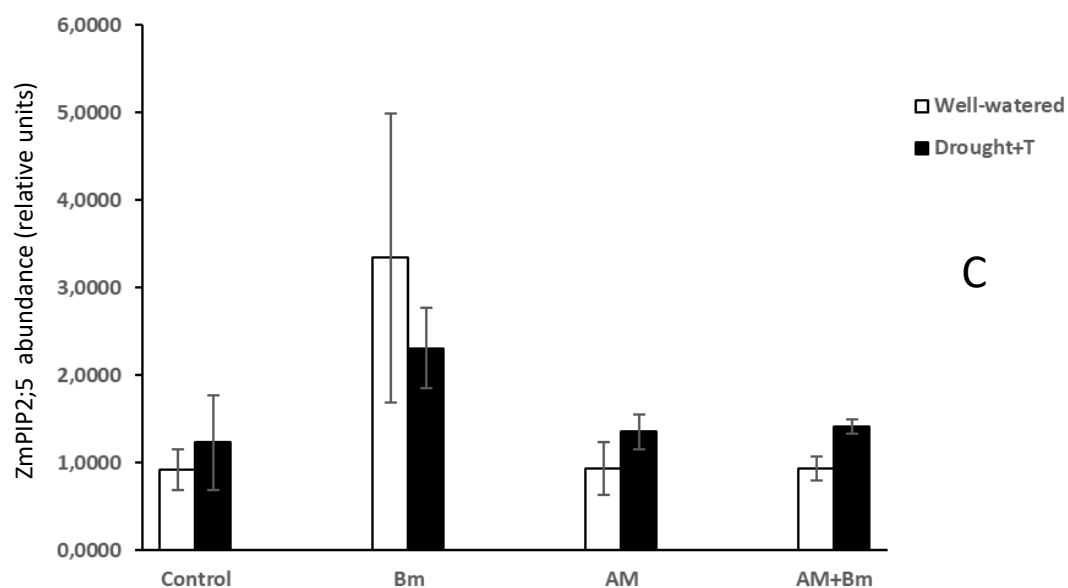

Supplement: Supplementary file 1 [file ijms-24-05193-s001.zip › Figure supplementary S1.pdf]

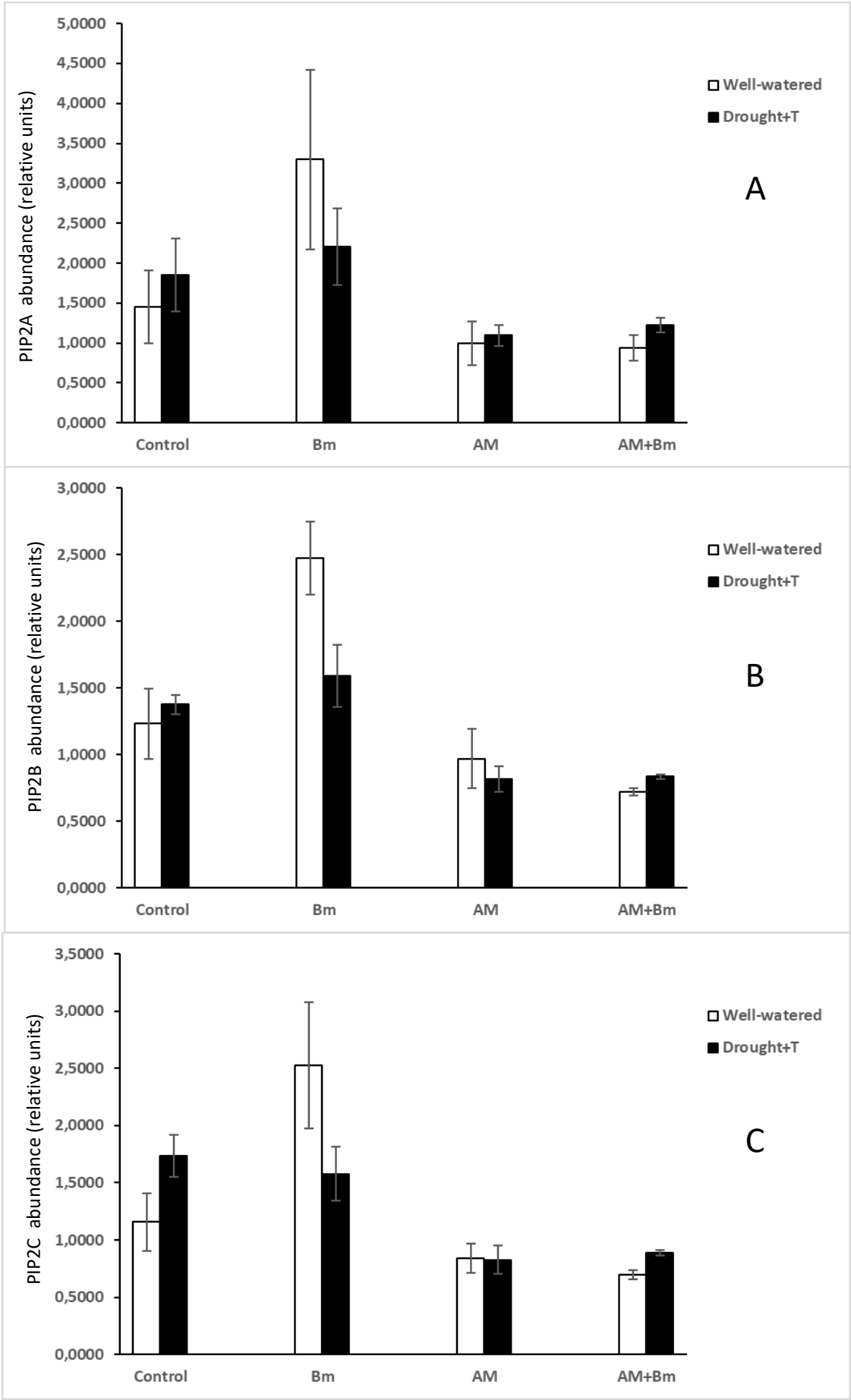

Supplement: Supplementary file 1 [file ijms-24-05193-s001.zip › Figure supplementary S2.pdf]
